# Sky Monitoring System for Flying Object Detection Using 4K Resolution Camera

**DOI:** 10.3390/s20247071

**Published:** 2020-12-10

**Authors:** Takehiro Kashiyama, Hideaki Sobue, Yoshihide Sekimoto

**Affiliations:** 1 Institute of Industrial Science, The University of Tokyo, 4-6-1 Komaba, Meguro-ku, Tokyo 153-8505, Japan; sekimoto@iis.u-tokyo.ac.jp; 2 School of Engineering, The University of Tokyo, 4-6-1 Komaba, Meguro-ku, Tokyo 153-8505, Japan; hideaki.s.0212@gmail.com

**Keywords:** flying object detection, drone, convolutional neural network, image processing

## Abstract

The use of drones and other unmanned aerial vehicles has expanded rapidly in recent years. These devices are expected to enter practical use in various fields, such as taking measurements through aerial photography and transporting small and lightweight objects. Simultaneously, concerns over these devices being misused for terrorism or other criminal activities have increased. In response, several sensor systems have been developed to monitor drone flights. In particular, with the recent progress of deep neural network technology, the monitoring of systems using image processing has been proposed. This study developed a monitoring system for flying objects using a 4K camera and a state-of-the-art convolutional neural network model to achieve real-time processing. We installed a monitoring system in a high-rise building in an urban area during this study and evaluated the precision with which it could detect flying objects at different distances under different weather conditions. The results obtained provide important information for determining the accuracy of monitoring systems with image processing in practice.

## 1. Introduction

In recent years, the use of drones and other unmanned aerial vehicles (UAVs) has expanded rapidly. These devices are currently used in various fields worldwide. In Japan, where natural disasters are a frequent occurrence, these devices are expected to be used for applications such as scanning disaster sites and searching for evacuees. In societies with shrinking populations, especially in areas with declining populations suffering from continuous labor shortages, there is hope that these devices can serve as new means for delivering medical supplies to emergency patients and food supplies to those without easy access to grocery stores.

However, there are growing concerns over these devices being misused for terrorism or other criminal activities. An incident occurred in April 2015 in Japan, when a small drone carrying radioactive material landed on the roof of the Prime Minister’s official residence. Moreover, there have been incidents where drones have crashed during large events or at tourist spots. In response to such incidents, legislation has been passed in Japan to impose restrictions on flying UAVs near sensitive locations such as national facilities, airports, and population centers and to set rules and regulations on safely flying these devices. However, this has not stopped the unauthorized use of drones by foreign tourists unaware of these regulations or users who deliberately ignore them. Hence, the number of accidents and problems continues to increase each year.

As the number of drones in use can only increase, it becomes important to ensure that the skies are safe. Therefore, a framework must be developed to prevent drones’ malicious use, such as for criminal activities or terrorism. Sky monitoring systems must address this important responsibility. Currently, methods using radar, acoustics, and RF signals to detect UAVs or radio-controlled aircraft [1] have been proposed. However, the systems [2,3,4,5] that use radar technology can impact the surrounding environment (such as blocking radio waves from nearby devices) and can only be used in limited areas. The method that uses acoustics [6] cannot detect a drone in a noisy urban environment, limiting the types of environments where the system can be installed. The methods in which drones are detected based on RF signals between the drone and operator [7] face the issue that they cannot detect autonomous control drones that do not transmit RF signals.

Methods employing images [8,9,10,11] have likewise been proposed, as we can see from the fact that image processing is used in various fields [12,13,14,15,16,17,18]. These methods have gained superiority, as they do not affect the monitoring targets or the surrounding environment and allow for systems to be built at a comparatively lower cost. It is also easy to miniaturize such systems, allowing them to be installed almost anywhere. Another benefit is that it becomes possible to use an indoor monitoring system to monitor an outdoor environment, reducing maintenance costs. Sobue et al. [8] and Seidaliyeva et al. [11] used background differencing to detect flying objects. Schumann et al. [9] used either background differencing or a deep neural network (DNN) to detect flying objects and used a separate convolutional neural network (CNN) model for categorization. Unlu et al. [10] proposed a framework in which the YOLOv3 [19] model was used to scan a wide area captured using a wide-angle camera to detect possible targets, and subsequently, a zoom camera was used to inspect the details of suspicious objects.

Although the aforementioned studies have explained the framework enough, the flying objects detected during the evaluation were large, and factors such as changes in the environment were not considered to a sufficient degree when evaluating their performance. Therefore, it is unclear under which conditions detection would succeed or fail in these methods, thereby making it difficult to determine whether they can be put to actual use. In this study, we propose a system capable of achieving a wide range of observations in real-time at a lower cost compared to existing systems using a single 4K resolution camera and YOLOv3, which is the state-of-the-art CNN. We installed the proposed system in a high-rise building located in the Tokyo metropolitan area, collected images that include flying objects, and evaluated the monitoring precision in changing weather and assuming various distances from the monitoring targets. It should be noted that the monitoring system was tested only during the day.

The remainder of this paper is organized as follows. We describe the proposed sky monitoring system in Section 2. The detection accuracy is presented in Section 3, and the results are discussed in Section 4. Finally, Section 5 concludes the study.

## 2. Proposed System

### 2.1. System Overview

We developed a sky monitoring system for use in urban areas that present a substantial risk concerning devices such as UAVs. The following restrictions must be assumed for use in an urban area:

- It is difficult to install equipment outdoors in high-rise buildings.
- It is difficult to use laser-based devices due to the Japanese Radio Act.
- It is difficult to use acoustic-based devices due to the high amount of noise in urban areas.
- It is difficult to monitor a wide area with a single system because of structures such as neighboring buildings.

Due to these restrictions, in this study, we installed a video camera indoors and developed a system that only uses image processing for monitoring. Using a video camera instead of special equipment, such as lasers, allowed the installation environment to be resolved and the system to be built at a reasonable cost. This also allowed us to install multiple systems.

In addition to monitoring nearby UAVs’ flight status, we decided to monitor flying objects in general. We assumed that the system would also be used for building management and would need to monitor the sky to prevent damage by birds. The system’s objective was to detect objects of a total length of approximately 50 cm or longer, which is the typical size of most general-purpose drones. The system was designed to monitor an area extending 150 m from it. This distance was selected for two reasons. First, UAV regulations within Japan permit flight only at an altitude of 150 m or lower. Second, Amazon has proposed a drone fly zone from 200 to 400 feet (61 to 122 m) for high-speed UAVs, and a fly zone up to 200 feet (61 m) for low-speed UAVs [20]. Although it would also be possible for a UAV used for malicious purposes (such as terrorism or criminal activity) to approach from an altitude of 150 m or higher, we considered that detecting small flying objects at a greater distance is not realistic when using only image processing. In addition, if the goal of drones is surreptitious photographing or dropping hazardous materials, the drones finally need to approach the facility. Therefore, we believe that the proposed system would be suitable for practical use under the aforementioned distance limitations.

### 2.2. System Configuration

**Figure 1:**
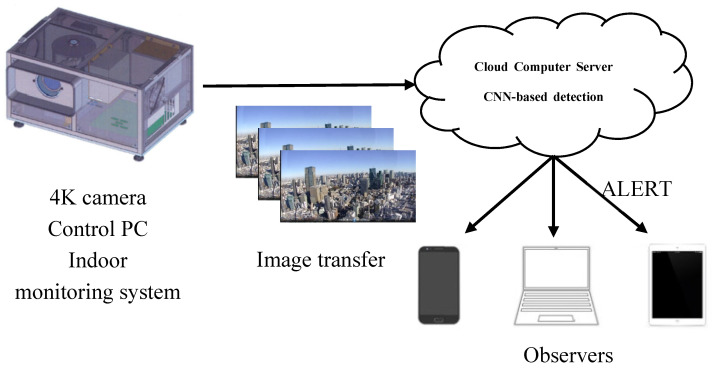
Proposed system configuration.

The proposed system was configured from an indoor monitoring system that includes a video camera and a control PC for transmitting the captured video, and a processing server that detects flying objects using a CNN. Figure 1 shows the proposed system configuration.

The monitoring system was equipped with a 4K video camera (which is the highest resolution offered by current consumer video cameras) to detect flying objects up to a distance of 150 m, and the widest angle is used to capture the footage of the sky to cover a wide area. It was assumed that the system would be installed indoors. Hence, we cannot ignore the fact that light would often be reflected from windows in typical office buildings, as blinds are opened and closed, or multiple lights are switched on. A covering hood was installed between the camera and window to reduce the impact of window reflections. The system was painted mostly black to reduce reflected light. This is a simple physical measure against reflected light, but it is essential for indoor installation.

A c5.4xlarge instance type processing server with 16 virtual CPUs was selected from Amazon EC2 as the processing server. In our proposed system, one 4K frame per second was sent from the server’s monitoring system for processing. This instance type was selected as it would be sufficient to process 4K images in real-time.

### 2.3. CNN-Based Detection

Recently, CNN, which represents a deep learning approach, has been applied in numerous computer vision tasks, such as image classification and object detection [21]. Many models [19,22,23] have been proposed and compared in terms of accuracy on the COCO [24] and KITTI [25] benchmark datasets. In this study, we use the YOLOv3 [19] model to detect flying objects. YOLOv3 extracts the features of an image by down-sampling the input image with filters of three sizes of 8, 16, and 32 to detect objects of different sizes. The training process uses the loss that is calculated based on both the objectness score calculated from bounding box coordinates (x, y, w, h) and the class score. The advantage of YOLOv3 is the high balance between processing speed and accuracy. Therefore, we presume that YOLOv3 would be most suitable for use in the security field, which requires both real-time processing and high detection accuracy. In 2020, YOLOv4 [26] and YOLOv5 [27] were developed. However, in consideration of stable operation, we adopted YOLOv3, which boasts abundant practical results.

A collection of images of airplanes, birds, and helicopters was used as data to train the model instead of drone images. This was done for two reasons. First, there is no publicly available dataset for use in CNN training. Second, it would likely be impossible to collect images of all types of drones, because as opposed to automobiles and people, they assume various shapes. Therefore, images of airplanes, birds, and helicopters were collected for model training and evaluation during this study.

The proposed system uses a 4K video camera to capture a wide view of the sky. A drone with a size of approximately 50 cm flying at a maximum distance of 150 m would span approximately 10 pixels in an image. If the system can detect airplanes, birds, and helicopters under such strict conditions, it is expected to be capable of detecting drones.

**Figure 2:**
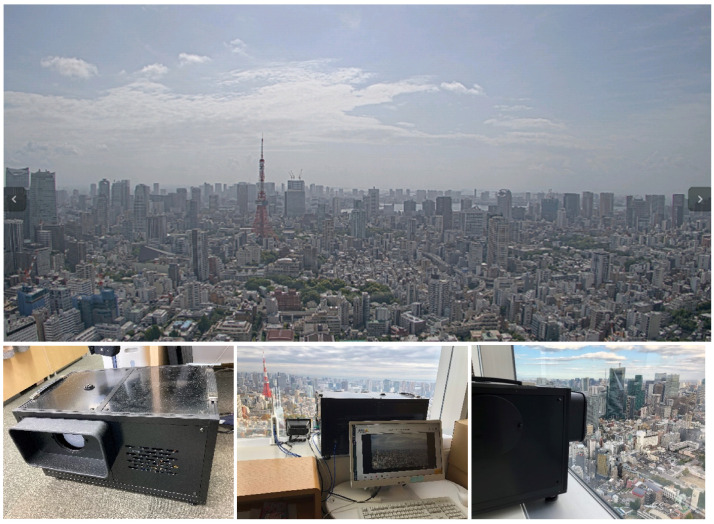
Image captured by the monitoring system and system installation.

The monitoring system was installed indoors on the 43rd floor of Roppongi Hills located in the center of Tokyo, to gather training data. Images of external flying objects were captured indoors through a window. Figure 2 shows an example of an image captured.

### 2.4. Input to Model

**Figure 3:**
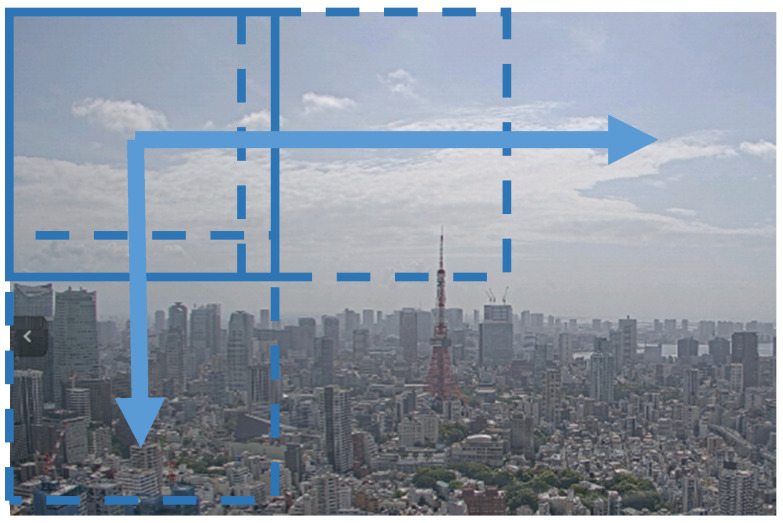
4K image input.

We determined that compressing 4K images and importing them into the YOLO model would not be an appropriate method to input high-resolution images of distant flying objects into the current model. Therefore, when processing the detection of flying objects on the processing server, 600 × 600 pixel squares were extracted from 3840 × 2160 4K images for input into the YOLO model. This is illustrated in Figure 3. These extracted images were partially duplicated, such that the precision of the detected objects would not decrease in the image boundary areas. Detection processes for each image were run in parallel on different virtual CPUs, allowing the detection process for the entire area of a 4K image to be completed within one second (i.e., in real-time).

### 2.5. CNN Model Training

**Figure 4:**
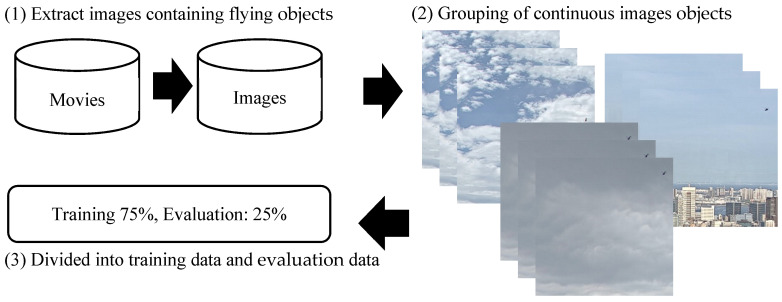
Separation of data for model training.

Images of flying objects were extracted from videos photographed by the proposed system. Therefore, continuous images were similar among frames, although not completely identical. When such continuous groups of images are randomly split into training and evaluation datasets, the training and evaluation datasets contain almost the same image. In this situation, the trained detection model is not accurately evaluated, resulting in the precision being evaluated highly. Therefore, first, image data were separated by each continuous frame group, as shown in Figure 4. Then, each group was randomly divided into training and evaluation datasets. Notably, 75% of the data was used to train the CNN model, while the remaining 25% was used for evaluation. Furthermore, the size of the flying object and weather were adjusted to be evenly distributed in this process.

For hyperparameters, when training the model, we used the default settings published by the developers of YOLOv3. Additional details are provided on the developer’s GitHub page [28]. Therefore, when training the model, basic data augmentation, such as flips, was performed. Furthermore, the brightness of the trained images was scaled between 0.3 and 1.2, and the number of trained images was expanded by approximately 10 times to increase robustness to changes in brightness due to changes in the time of day and weather.

## 3. Evaluation

### 3.1. Categorization of Data for Evaluation

The purpose of the evaluation process was to determine the effect of differences in the type of weather or sizes of flying objects on flying objects’ detection precision. The images gathered, as described in Section 2.3, were categorized by conditions. The collected image data contained 1392 helicopters, 190 airplanes, and 74 birds. It should be noted that the background images did not include buildings and trees, but only the sky, as most flying objects were airplanes and helicopters.

**Figure 5:**
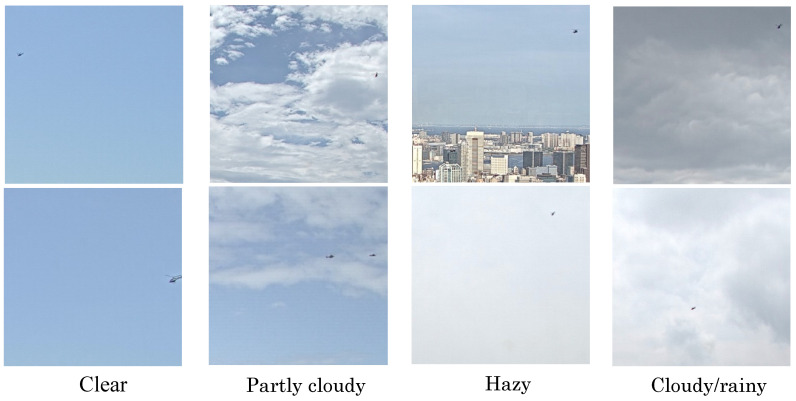
Categorization of data by type of weather.

The images were categorized by four types of weather: clear, partly cloudy, hazy, and cloudy/rainy. The images were categorized by eye, based on whether they contained clouds with contours, haze, blue skies, or rain clouds (dark clouds). Figure 5 lists the results of categorizing the images according to the type of weather.

The images were then sorted into six categories corresponding to the sizes of the flying objects based on the number of horizontal pixels shown in the image: SS (less than 12 pixels), S (12 to 16 pixels), M (17 to 22 pixels), L (23 to 30 pixels), LL (31 to 42 pixels), and 3L (43 pixels or more). These categories can also be expressed as the distance in meters assuming a drone of approximately 50 cm size (SS: 150 m or greater, S: 110 m to less than 150 m, M: 75 m to less than 110 m, L: 55 m to less than 75 m, LL: 38 m to less than 55 m, and 3L: less than 38 m).

Table 1 lists the results of categorizing the images. A few images were categorized as clear, and many were categorized as hazy, with 253 clear, 433 partly cloudy, 601 hazy, and 368 cloudy/rainy images. There were comparatively few images categorized as SS, S, and 3L, but a roughly equivalent number categorized as all other sizes, with 58 SS, 182 S, 368 M, 488 L, 395 LL, and 152 3L images.

### 3.2. Evaluation Indicators

A precision-recall (PR) curve with precision on the vertical axis and recall on the horizontal axis was used for evaluation. Rectangular bounding boxes are generally used for estimation when detecting objects. A fixed threshold was used in numerous studies to evaluate the intersection over union (IoU) calculated from the estimated and actual bounding boxes. However, owing to the extremely small size of the detected objects in this study, a large proportion of measurement errors would be included for the flying objects in the bounding boxes. This is due to the blurriness caused by the lens or misalignment resulting from the human annotation. Therefore, we determined that a flying object was detected during the evaluation process if the actual and estimated bounding boxes overlapped even slightly.

**Figure 6:**
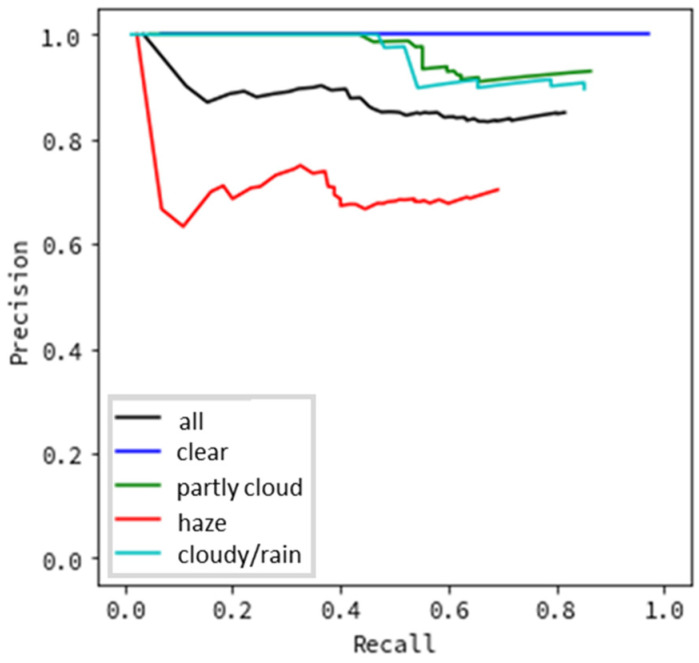
Detection precision by type of weather (including categorization).

**Figure 7:**
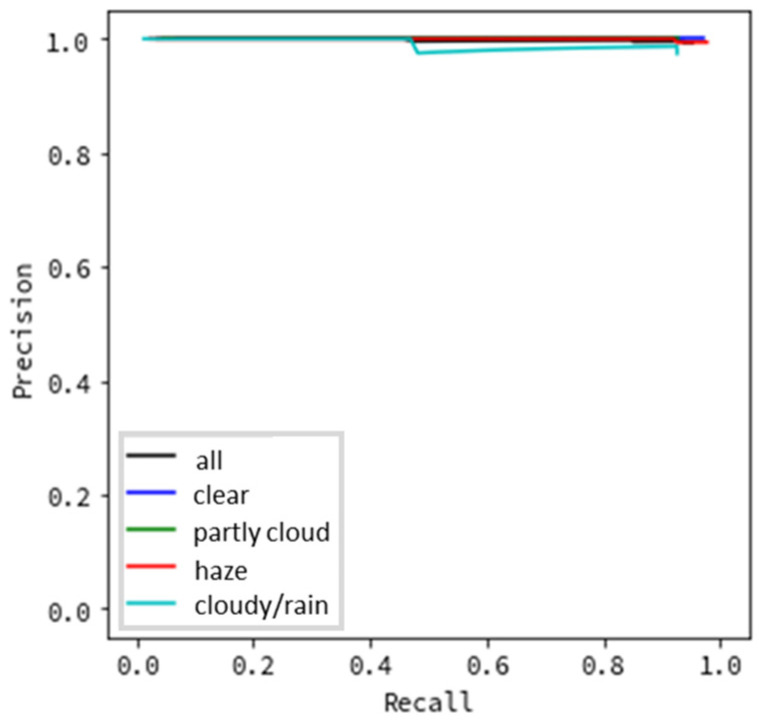
Detection precision by type of weather (not including categorization).

Figure 6 and Figure 7 present the results of evaluating the precision based on the type of weather. In Figure 6, images where a flying object was successfully detected and subsequently successfully categorized as a helicopter, bird, or airplane were considered as true positives. The remainder (where the flying object was successfully detected but categorized incorrectly) was false positive. In Figure 7, images where a flying object was successfully detected (even if categorization failed) were evaluated as true positives, while the remainder was evaluated to be false positive. This was conducted under the assumption that even succeeding only at detection would demonstrate that the system offers the minimum required functionality for monitoring flying objects.

**Figure 8:**
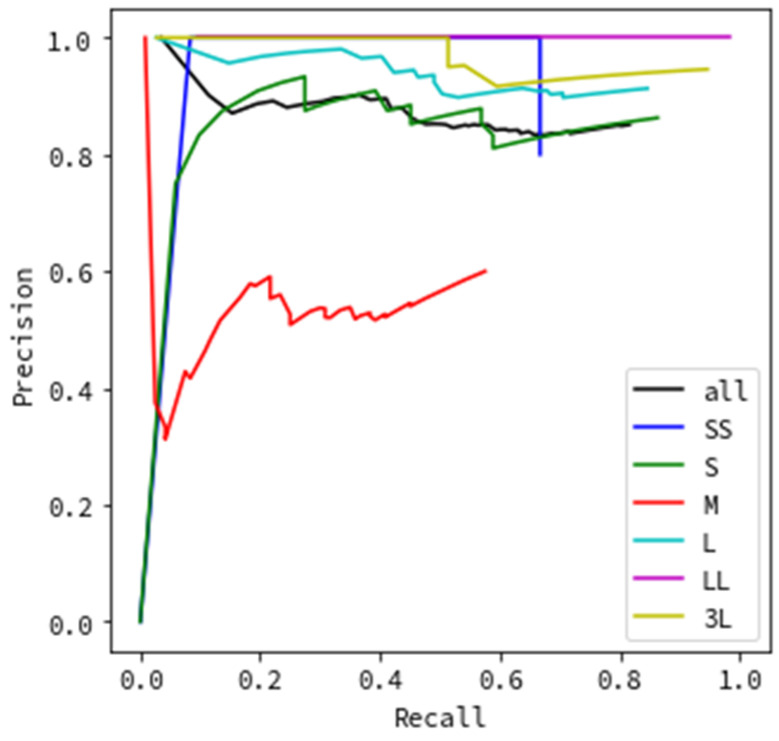
Detection precision by flying object size (including categorization).

**Figure 9:**
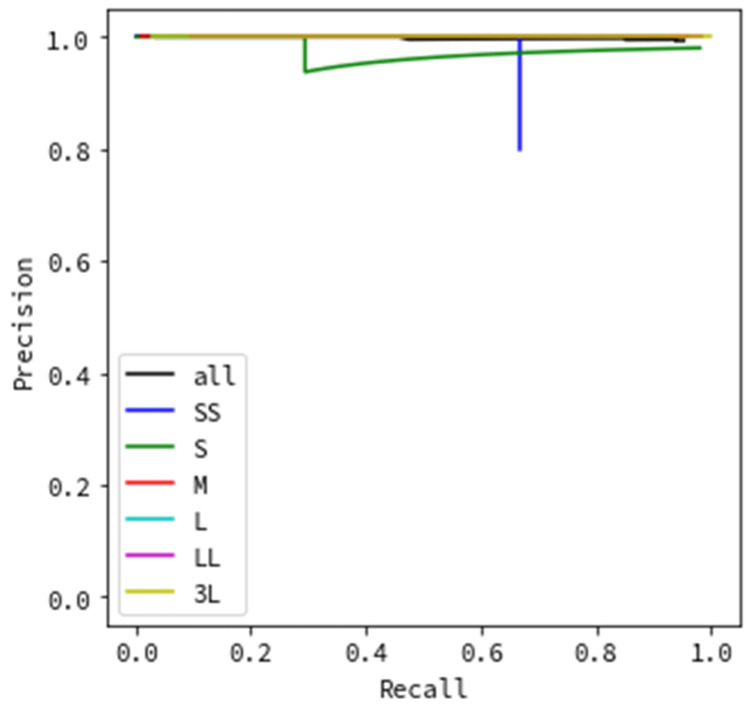
Detection precision by flying object size (not including categorization).

Figure 8 and Figure 9 show the results of evaluating the precision based on the flying object’s size. Similar to the evaluation according to the type of weather, in Figure 8, images where a flying object was successfully detected and then successfully categorized as a helicopter, bird, or airplane were evaluated as true positives, while the remainder was considered false positive. Figure 9 shows that images where a flying object was successfully detected (even if categorization failed) were evaluated as true positives, while the remainder was considered false positive.

All evaluation data results are depicted as black lines in Figure 6, Figure 7, Figure 8 and Figure 9, while the results for each type of weather are color-coded.

## 4. Discussion

We first discuss the results obtained for the precision evaluation based on the type of weather. Figure 6 shows excellent results for clear weather, with almost no false detections. The results for partly cloudy weather (when there are some clouds in the sky) and cloudy/rainy weather (when the entire sky is covered in clouds) are also satisfactory, although not as good as during clear weather. When the recall is 0.8, the results indicate a precision above 0.8. In contrast, the results are worse for hazy weather. When the recall is 0.6, the precision is approximately 0.6. We believe that this is due to the haze blurring the contours of flying objects. In this evaluation, because we targeted detecting flying objects such as helicopters and aircraft, which may be anywhere from several hundred meters to several kilometers away from the monitoring system, the effect may significantly influence detection accuracy. If we consider drones, the flight distance will be remarkably closer than the current targets. Therefore, the accuracy decrease due to hazy weather may be small, which must be investigated in the future. Notably, Figure 6 shows that although clouds and rain lower the overall brightness of images and make it difficult to detect objects by sight, no decrease in precision is observed in the monitoring system. Hence, the noise of the flying object’s appearance, and not the change in the brightness of the sky, affects its detection accuracy. In contrast, Figure 7 indicates excellent results in all types of weather, with a precision value of nearly 1.0 for all recall values. This demonstrates that flying object categorization mistakes, rather than detection omissions, have a significant influence in lowering the precision and recall. If the system obtains human support for the classification task, the systems based on image processing can contribute significantly to sky monitoring.

Next, we discuss evaluating the precision based on the sizes of flying objects in the images. The results in Figure 8 indicate excellent results for L, LL, and 3L, which correspond to large sizes; when the recall is 0.8, the precision is above 0.8. In contrast, SS, S, and M, which have relatively small sizes, yield different results for each size. The result of the SS size category is an unnatural value. In the evaluation process, the evaluation data were separated into six categories by size, and the number of images per size category was different (SS: 58, S: 82, M: 368, L: 488, LL: 395, and 3L: 152). Focusing on SS, the number of images is very small. This implies that the precision for SS-sized objects was likely not calculated accurately. However, it also shows the capability of detecting even the smallest flying objects, which should be investigated in the future. The S results in a comparatively good PR curve, comparable to the results for large flying objects (L, LL, 3L). When the recall is 0.8, the precision achieved is above 0.8. This size corresponds to a distance of 110 to 150 m for a drone of maximum length (roughly 50 cm), and it is the minimum size of the monitoring system’s observation target. This indicates that the monitoring system using image processing achieves sufficient detection accuracy that satisfies the requirement for a distance of 150 m, as shown in Section 2.1. The M size category shows remarkably poor results that are worse than those of all other size categories. We attribute this to the hazy weather often occurring for this size of an object compared with others. From the values in Table 1, for the M-size images, 246 images, which comprise more than half of the evaluation data of 368 images, are images captured in hazy weather. Hence, it is considered that the accuracy was reduced due to the influence of the weather and not size. Thus, we can conclude that high detection accuracy is achieved for flying object sizes S and above, excluding hazy weather. For the trials without categorizations, similarly to Figure 7, Figure 9 portraying the effect of flying object size shows particularly excellent results with an achieved precision value of nearly 1.0 for all recall values. This indicates that flying object categorization mistakes, rather than detection omissions, significantly impact the lower precision and recall by size, as shown in Figure 8.

In summary, the results indicate that the precision is poor during hazy weather, and the difference in accuracy depending on the object size is not large if it is S or above. Overall, except for hazy weather, the system maintains a precision value of approximately 0.8 when the recall is 0.8, confirming that it offers sufficient precision to function as a monitoring system. Therefore, further improvement of the algorithm to achieve better performance in hazy weather is required for actual use. The results also confirm that the detection precision is affected by flying object categorization mistakes rather than detection omissions. Under the experiment in this study, if we ignore flying objects’ classification, almost all flying objects are detected in this study. Assuming that there are not many flying objects in the sky, unlike pedestrian detection in the city, even if the classification accuracy is poor, we believe that it can contribute to real scenes as a monitoring system. However, the images of the flying objects used during this study contain only the sky as a background. Images captured in an urban area are likely to include buildings behind flying objects due to building density in the area. This would create a more complicated background and make it significantly more difficult to detect categories. Therefore, the precision of this system must be verified under a range of conditions for use in urban areas.

Furthermore, although not examined in this study, capturing clear images is an important issue in monitoring systems that use image processing. When installing the system externally, the camera lens becomes dirty due to rain, insects, and dust, and it is not possible to obtain clear images. Even when installed indoors, it is affected by dirt on the glass in front of the monitoring system. If the object is relatively large, such as a pedestrian or a car, this will not have a significant negative impact. However, for flying objects, where the detection target is extremely small, the detection accuracy is substantially affected. Because physical solutions for this challenge are cumbersome, software-based methods are necessary to solve these problems for practical use.

## 5. Conclusions

We developed a sky monitoring system that uses image processing. This system consists of a monitoring system and a cloud processing server. The monitoring system captures a wide area of the sky at high resolution using a 4K camera and transfers the frame image to the server every second. The processing server uses the YOLOv3 model for flying object detection. In this process, real-time processing is realized by the parallel processing of multiple cropped images. We installed the monitoring system in a high-rise building in the Tokyo metropolitan area and collected CNN model training and evaluation data to evaluate the system’s detection precision.

Existing research has employed the CNN model; however, the accuracy was not analyzed for each condition, and it was generally insufficient for practical use. Further, there is no simple system that combines a single 4K camera with the latest CNN to date. We designed a real-time monitoring system prototype using the latest CNN model and 4K resolution camera and demonstrated the detection accuracy of flying objects with various object sizes and weather conditions.

We found that detection accuracy is significantly reduced in hazy weather. From this result, we found that the blurring of the flying object’s shape in the haze and the decrease in brightness in rainy or cloudy weather influence the decrease in accuracy. The flying object’s size was replaced by the distance of the flying object in this study, and we show that a flying object with a pixel-sized image, assuming a 50 cm drone separated by 150 m, can be detected with high accuracy. Moreover, if we omit the classification accuracy, we find that almost all flying objects can be detected under all weather conditions and sizes considered in this experiment. From these results, the surveillance system using image processing is expected to contribute to sky surveillance, as the cost of human support in classification is low in an environment where there are few objects in the sky.

However, the monitoring system has several limitations: it was verified only during the daytime in this experiment, and we did not consider the effects of dirt and dust on the camera lens on the image. Moreover, the evaluation data targeted long-distance aircraft and helicopters as flying objects and did not include drones that fly relatively short distances. In the future, we plan to evaluate the monitoring accuracy in further detail by collecting evaluation data, including the above conditions. In this research, we will improve the proposed monitoring and develop a system to detect light emitted from LEDs installed on drones and a system that uses a comparatively inexpensive infrared projector for the detection of drones at night.

## 6. Patents

Japan patent: JP6364101B.

## Figures and Tables

**Table 1:** Number of images by size and weather.

Image Size	Estimated Distance	Clear	Partly Cloudy	Hazy	Cloudy/Rainy
SS (up to 11 pixels)	From 150 m	7	11	19	21
S (up to 16 pixels)	110–150 m	5	57	68	52
M (up to 22 pixels)	75–110 m	12	69	246	41
L (up to 30 pixels)	55–75 m	47	147	149	145
LL (up to 42 pixels)	38–55 m	77	128	99	91
3L (from 43 pixels)	Up to 38 m	105	21	20	19
SUM		253	433	601	369

## References

[1] Taha B., Shoufan A. (2019). Machine Learning-Based Drone Detection and Classification: State-of-the-Art in Research. IEEE Access.

[2] Samaras S., Diamantidou E., Ataloglou D., Sakellariou N., Vafeiadis A., Magoulianitis V., Lalas A., Dimou A., Zarpalas D., Votis K. (2019). Deep Learning on Multi Sensor Data for Counter UAV Applications-A Systematic Review. Sensors.

[3] Mendis G.J., Randeny T., Wei J., Madanayake A. Deep learning based doppler radar for micro UAS detection and classification. Proceedings of the MILCOM 2016—2016 IEEE Military Communications Conference.

[4] Ganti S.R., Kim Y. Implementation of detection and tracking mechanism for small UAS. Proceedings of the International Conference on Unmanned Aircraft Systems (ICUAS).

[5] Kwag Y., Woo I., Kwak H., Jung Y. Multi-mode SDR radar platform for small air-vehicle Drone detection. Proceedings of the CIE International Conference on Radar (RADAR).

[6] Hommes A., Shoykhetbrod A., Noetel D., Stanko S., Laurenzis M., Hengy S., Christnacher F. Detection of Acoustic, Electro-Optical and Radar Signatures of Small Unmanned Aerial Vehicles. Proceedings of the SPIE Security + Defence.

[7] Ezuma M., Erden F., Anjinappa C.K., Ozdemir O., Guvenc I. Micro-UAV Detection and Classification from RF Fingerprints Using Machine Learning Techniques. Proceedings of the IEEE Conference on Aerospace.

[8] Sobue H., Fukushima Y., Kashiyama T., Sekimoto Y. Flying object detection and classification by monitoring using video images. Proceedings of the 25th ACM SIGSPATIAL International Conference on Advances in Geographic Information Systems.

[9] Schumann A., Sommer L., Klatte J., Schuchert T., Beyerer J. Deep cross-domain flying object classification for robust UAV detection. Proceedings of the IEEE International Conference on Advanced Video and Signal Based Surveillance (AVSS).

[10] Unlu E., Zenou E., Riviere N., Dupouy P.E. (2019). Deep learning-based strategies for the detection and tracking of drones using several cameras. IPSJ Trans. Comput. Vis. Appl..

[11] Seidaliyeva U., Akhmetov D., Ilipbayeva L., Matson E.T. (2020). Real-Time and Accurate Drone Detection in a Video with a Static Background. Sensors.

[12] Chato P., Chipantasi D.J.M., Velasco N., Rea S., Hallo V., Constante P. Image processing and artificial neural network for counting people inside public transport. Proceedings of the IEEE Third Ecuador Technical Chapters Meeting.

[13] Ahmed S., Huda M.N., Rajbhandari S., Saha C. (2019). Pedestrian and Cyclist Detection and Intent Estimation for Autonomous Vehicles: A Survey. Appl. Sci..

[14] Riyazhussain S., Lokesh C.R.S., Vamsikrishna P., Rohan G. Raspberry pi controlled traffic density monitoring system. Proceedings of the International Conference on Wireless Communications, Signal Processing and Networking (WiSPNET).

[15] Zhang F., Li C., Yang F. (2019). Vehicle detection in urban traffic surveillance images based on convolutional neural networks with feature concatenation. Sensors.

[16] Fedorov A., Nikolskaia K., Ivanov S., Shepelev V., Minbaleev A. (2019). Traffic flow estimation with data from a video surveillance camera. J. Big Data.

[17] Maeda H., Sekimoto Y., Seto T., Kashiyama T., Omata H. (2018). Road Damage Detection and Classification Using Deep Neural Networks with Smartphone Images. Comput.-Aided Civ. Infrastruct. Eng..

[18] Arshad B., Ogie R., Barthelemy J., Pradhan B., Verstaevel N., Perez P. (2019). Computer Vision and IoT-Based Sensors in Flood Monitoring and Mapping: A Systematic Review. Sensors.

[19] Redmon J., Farhadi A. (2018). Yolov3: An incremental improvement. arXiv.

[20] Amazon PrimeAir https://www.amazon.com/Amazon-Prime-Air/b?ie=UTF8&node=8037720011.

[21] LeCun Y., Bengio Y., Hinton G. (2015). Deep learning. Nature.

[22] Liu W., Anguelov D., Erhan D., Szegedy C., Reed S., Fu C.-Y., Berg A.C. (2016). Ssd: Single shot multibox detector. Proceedings of the European Conference on Computer Vision.

[23] Ren S., He K., Girshick R., Sun J. Faster r-cnn: Towards real-time object detection with region proposal networks. Proceedings of the Advances in Neural Information Processing Systems.

[24] COCO (Common Objects in Context). https://cocodataset.org/.

[25] Geiger A., Lenz P., Stiller C., Urtasun R. (2013). Vision meets robotics: The kitti dataset. Int. J. Robot. Res..

[26] Bochkovskiy A., Wang C.Y., Liao H.Y.M. (2020). YOLOv4: Optimal Speed and Accuracy of Object Detection. arXiv.

[27] Ultralytics https://github.com/ultralytics/yolov5.

[28] YoloV3 GitHub https://pjreddie.com/darknet/yolo/.

